# Finding a Needle in the Haystack: The Costs and Cost-Effectiveness of Syphilis Diagnosis and Treatment during Pregnancy to Prevent Congenital Syphilis in Kalomo District of Zambia

**DOI:** 10.1371/journal.pone.0113868

**Published:** 2014-12-05

**Authors:** Bruce A. Larson, Deophine Lembela-Bwalya, Rachael Bonawitz, Emily E. Hammond, Donald M. Thea, Julie Herlihy

**Affiliations:** 1 Center for Global Health and Development, Boston University, Boston, MA, United States of America; 2 Zambia Center for Applied Health Research and Development, Lusaka, Zambia; 3 Department of Pediatrics, Boston Medical Center, Boston, MA, United States of America; Agence de Médecine Préventive, France

## Abstract

**Background:**

In March 2012, The Elizabeth Glaser Pediatric AIDS Foundation trained maternal and child health workers in Southern Province of Zambia to use a new rapid syphilis test (RST) during routine antenatal care. A recent study by Bonawitz et al. (2014) evaluated the impact of this roll out in Kalomo District. This paper estimates the costs and cost-effectiveness from the provider's perspective under the actual conditions observed during the first year of the RST roll out.

**Methods:**

Information on materials used and costs were extracted from program records. A decision-analytic model was used to evaluate the costs (2012 USD) and cost-effectiveness. Basic parameters needed for the model were based on the results from the evaluation study.

**Results:**

During the evaluation study, 62% of patients received a RST, and 2.8% of patients tested were positive (and 10.4% of these were treated). Even with very high RST sensitivity and specificity (98%), true prevalence of active syphilis would be substantially less (estimated at <0.7%). For 1,000 new ANC patients, costs of screening and treatment were estimated at $2,136, and the cost per avoided disability-adjusted-life year lost (DALY) was estimated at $628. Costs change little if all positives are treated (because prevalence is low and treatment costs are small), but the cost-per-DALY avoided falls to just $66. With full adherence to guidelines, costs increase to $3,174 per 1,000 patients and the cost-per-DALY avoided falls to $60.

**Conclusions:**

Screening for syphilis is only useful for reducing adverse birth outcomes if patients testing positive are actually treated. Even with very low prevalence of syphilis (a needle in the haystack), cost effectiveness improves dramatically if those found positive are treated; additional treatment costs little but DALYs avoided are substantial. Without treatment, the needle is essentially found and thrown back into the haystack.

## Introduction

The Zambian Ministry of Health's guidelines for antenatal care (ANC) recommend that all pregnant women be tested for HIV and syphilis during their first visit for antenatal care. While HIV testing has been solidly integrated into ANC in Zambia, syphilis testing has not. For example, 94% of all women accessing antenatal care were also tested for HIV by 2012 [1]. For syphilis testing, a recent study of ANC service delivery reported that 50% of facilities evaluated nationwide (n = 1299) did not provide syphilis testing [2]. Another recent study reported that fewer than 50% of pregnant women received a syphilis test in facilities with testing capabilities (e.g., the service was in principle available) [3].

In March 2012, The Elizabeth Glaser Pediatric AIDS Foundation trained maternal and child health workers in four districts in the Southern Province of Zambia to use a new point-of-care and rapid syphilis test (RST), BIOLINE 3.0, during routine antenatal care. While the existing standard of care was the *rapid plasma regain* (RPR) test, the RST is simple to use compared to RPR testing [4]. A recent study by Bonawitz et al. (2014) evaluated the impact of this training and roll out of the RST, using a pre-post study design, in 18 randomly selected Ministry of Health ANC clinics in Kalomo District [5]. At baseline, prior to the RST roll out, only 10% of women received a syphilis test (using the RPR test). In the 12-month follow up period after the introduction of RST training, 62% of women attending their first ANC visit received an RST.

In this study, we first estimate the unit cost of syphilis screening with the recently introduced RST and resultant treatment. Second, we use these unit costs to estimate program costs from the provider's perspective, in this case the Ministry of Health, of implementing syphilis screening using the new RST in public clinics. And third, we use information on costs, patient care during the evaluation study, and estimates of adverse birth outcomes (ABOs) from active but untreated syphilis to evaluate the cost effectiveness of syphilis screening as part of the first ANC visit during pregnancy (as compared to no screening).

Previous research suggests that syphilis testing and treatment for pregnant women is likely to be “cost-effective” [3], [6]–[8]. The studies have been modeling exercises, in the sense that they consider a range of assumptions regarding prevalence, test quality (sensitivity and specificity), adherence to guidelines (proportions tested, treated, etc.), general international prices for commodities (test kits, penicillin, etc.), and potential impacts/benefits beyond the direct benefits of avoiding adverse birth outcomes during pregnancy. The analysis presented here complements these previous studies by using costs in Zambia, prevalence as observed during the evaluation study, and focusing impacts on adverse birth outcomes. Given the recent roll out of RSTs in Kalomo District and plans for larger-scale roll out in Zambia, the analysis presented in this paper can be used to assist with planning for, and evaluating, an expanded roll out.

We originally intended to evaluate cost-effectiveness of RSTs compared to RPR testing. However, because only 10% of women who should have been tested were tested with the standard of care technology (RPR testing) during the study baseline, the comparison would essentially be irrelevant. With only 10% receiving an RPR test, costs were very low but little was achieved. Although RPR testing has been the standard of care for some time, the very infrequent use of the RPR test eliminated this approach as a relevant comparison for this analysis.

## Methods

### Unit costs for testing and treatment

We estimated RST, and for perspective RPR, costs based on materials used per test. Prices for materials were based on prices provided in Zambia's Medical Stores Limited 2010 catalogue, which were then inflated using Zambia consumer price inflation index to 2012 values (CPI figures from the IMF World Economic Outlook Database), and the invoice from the company providing the RSTs during the evaluation study (Melcome Pharmaceuticals Limited, invoice from March 2013, with prices relevant in 2012). See File S1 for details.

A health worker (e.g., a nurse) completes an RST with the patient during an antenatal care visit (ideally her first visit for antenatal care). Multiple activities take place during an ANC visit. Owing to the simplicity of the RST, little health worker time is actually used during an ANC visit to complete the syphilis test. Thus, in practice, the additional labor time to perform the RST as part of an ANC visit is likely to be minor. However, time is needed nonetheless to explain why the syphilis test is recommended and to obtain consent. We follow Kuznik et al. (2013) and include 17.5 minutes of additional nurse time for syphilis testing [3]. For patients who test positive, we also add an additional 5 minutes of time to prepare and complete one injection of penicillin.

Guidelines prescribe three doses of penicillin over time following a positive syphilis test in pregnant women. However, one dose administered 30 days prior to delivery is considered protective of the newborn, decreasing risk of an adverse neonatal outcome to that of a mother without syphilis infection [9]. Since women in Zambia typically present for ANC care during their 20–24th week of gestation and few present in their final month ([10]), if a woman is treated, we assume here that a woman receives at least one dose of penicillin, that it is received more than 30 days before delivery, and that it reduces risks of adverse birth comes (ABOs) to that observed for those infants born to mothers without syphilis infection.

Because this analysis is focused on diagnosis and treatment to avoid ABOs from untreated syphilis, we focus only on impacts in terms of reduced ABO risks from the first injection of penicillin. While two additional injections are needed to treat fully the mother's syphilis, designed to be received as part of follow up ANC visits (not separate visits just for the injection), we exclude benefits beyond the primary reduction in ABOs due to the treatment during pregnancy. Thus, we also exclude costs (or perhaps cost savings) that are unrelated to ABOs (e.g., reduction in future syphilis transmission, woman's own health).

In addition to the cost of basic test commodities, we included the cost of health worker training (on syphilis, syphilis policy, and testing with RSTs) provided during the RST roll out in Southern Province. Both RPR testing and the newer RSTs were included in the training activities. Training costs were based on a 4-day curriculum with 46 participants and 10 staff (7 medical officers, 3 nurses) providing the training. We included salaries, participant per diems, transportation, meals, lodging, and other minor expenses. We used a 3% annual discount rate to estimate the monthly equivalent cost of the training per nurse, and assumed a useful life for the training of two years (given high turnover among ANC staff). Based on program records from Boston University PMTCT Integration Project, on average 36 new patients present for their first ANC visit monthly at the evaluation study health facilities in Kalomo District. With one nurse per site participating in the training activities, we divide the estimate of training costs per month by 36 to estimate the training cost per new ANC patient.

In sum, the unit costs for testing include:

- The RST;
- Other materials per test (e.g., gloves, alcohol);
- Health worker time to complete the test (including patient information and consenting);
- Training for health workers.

And the unit costs for treatment (per injection of penicillin) include:

- The dose of penicillin;
- Other materials used for the injection;
- Health worker time for the injection.

The focus here is on the cost of syphilis testing and treatment, as an additional service, at public clinics that already exist. Thus, clinic fixed costs (buildings, utilities, maintenance, security, etc.) are not relevant for the analysis. A plaster (bandage) could be included in material costs, but they are not necessarily used and costs are minor.

### RST positives, test sensitivity and specificity, and syphilis prevalence

The 2.8% of mothers who tested positive based on the RST during the evaluation study forms the starting point for this analysis. We then consider the range of possible RST sensitivities and specificities and prevalence that could be consistent with 2.8% RST positives. If the test is perfect, then true prevalence is also 2.8%. If the test specificity falls to 98%, which is still a very high specificity, true prevalence is only 0.8335% (a 29% positive predictive value). See [11] for the details linking test prevalence to true prevalence based on test sensitivity and specificity.

In addition, the RST is a treponemal test; that is, it measures the presence of specific antibodies against *Treponema pallidum*, the causative agent of syphilis. Individuals who have active syphilis as well as those previously treated and cured will test positive using the RST. Based on previous literature [7], we assume as a base case that 80% of women who are RST positive have active syphilis. Therefore, the 2.8% prevalence observed during the evaluation period translates into 0.8335% ‘true positives’ (defined as active and previously treated), and 0.667% with active syphilis.

### Costs of syphilis diagnosis and treatment and scenarios evaluated

A decision analytic model (similar to [3] for syphilis testing and [12] for malaria testing) was used to evaluate the expected costs of syphilis screening and treatment per 1,000 women presenting for antenatal care under a variety of scenarios. See File S2 for all details. In this model, we defined six branches as follows: (1) not tested/not treated; (2) not tested/treated; (3) tested/positive/treated;(4) tested/positive/not treated; (5) tested/negative/treated; (6) tested/negative/not treated. Each patient then falls into one of these six possible branches. The proportion of patients managed in each branch is based, as a base case, on how patients were actually managed after the RST roll out in Kalomo District. As reported in the evaluation study, 62% received an RST and 10.4% of those tested and positive were treated. All patients who were not tested were not treated, and no patients who were tested and found negative were treated. The costs for each branch are then simply the number of patients managed in each branch (1,000 times the proportion in each branch) multiplied by the medical services received (testing or not, treatment or not). The cost per 1,000 patients presenting for ANC care are then simply the sum of the costs for each branch. Table 5 shows how the basic model is organized in Excel.

For this analysis, we use as the base case the proportion testing positive during the evaluation study (2.8%), and 98% sensitivity and specificity of the RST. Note that the 2.8% proportion testing positive, which is consistent with 0.8% ‘true positive’ prevalence on the test and 0.6% active syphilis prevalence, is substantially less than the 8.3% syphilis prevalence used in a recent modeling exercise [3].

Each run of the model is based on a number of detail assumptions (how patients are managed, prevalence, costs, etc.). To simplify the discussion of the results, and to focus attention on a few key issues, we use the term “scenario” to represent a full set of assumptions for each run of the model (each estimate of cost per 1,000 patients). We focus on three main scenarios.

1. The base case, which uses our estimates of unit costs for diagnosis and treatment and information on patient management as observed during the evaluation study (62% tested, 2.8% positive, 10.4% of positives treated). For short, we will call this Scenario ES for evaluation study.
2. The next scenario is the same as Scenario ES except that all patients tested and found positive are actually treated (62% tested, 2.8% positive, 100% of positives treated). We call this Scenario ES + All Positives Treated.
3. The third main scenario is that all patients are managed according to ANC guidelines (100% tested, 2.8% positive, 100% of positives treated). We call this Scenario ES + Guidelines.

In addition to these three scenarios, we consider the sensitivity of these results to changes in key parameters in the model, such as prevalence, other patterns of patient management, staff time to complete tests, and training costs.

### Adverse birth outcomes (ABOs) and disability-adjusted life years (DALYs)

For this analysis, we use rates of adverse birth outcomes for women with and without syphilis during pregnancy—stillbirth/fetal loss; neonatal death; low-birth weight; clinical evidence of syphilis—reported in a recent meta-analysis [13]. We focus our analysis on two primary adverse birth outcomes: stillbirth/fetal loss; and neonatal death. In sum, from the meta-analysis, 7.6% of women without syphilis and 37.9% of women with syphilis are estimated to have these two ABOs.

Prior evidence indicates that if women with syphilis are treated with one dose of penicillin during pregnancy, then their ABO risks are same as for women without syphilis [9]. Therefore, we assume in this analysis 30.3% fewer (37.9%–7.6%) of these ABOs for women with active syphilis who receive at least one dose of penicillin.

Life expectancy at birth in Zambia is estimated at 55 years in 2011 [14]. With a 3% discount rate, disability adjusted life years lost for 55 years is 26.93 (see [15] for details). For every stillbirth/fetal loss or neonatal death, which essentially means full life expectancy at birth is lost, we therefore assume that 26.93 DALYs are lost. With 30% fewer of these ABOs per pregnant women with active syphilis treated during pregnancy, 0.30*26.93 = 8.07 DALYs are avoided per pregnant women with syphilis who is treated.

Two additional ABOs are excluded from this analysis, low birth weight/prematurity and clinical evidence of syphilis [7]. Based on the rates for these ABOs [13] and disability weights of 0.106 for low birth weight and 0.316 for congenital syphilis [7], an additional 1.39 DALYs would be avoided from these conditions due to treatment of a pregnant women with active syphilis (1.28 from syphilis, 0.17 from low birth weight). However, in practice it is difficult to differentiate prematurity from low birth weight. In addition, congenital syphilis is the term generally used for mother to child (MTC) transmission of syphilis, but it encompasses varying clinical manifestations in the infant or young child. While benefits from treatment, in terms of reduced mother-to-child transmission of syphilis for surviving infants, are real and substantial, the DALYs lost from such conditions are relatively small compared to DALYs lost from mortality. As a result, excluding them from the analysis has little impact on the basic cost-effectiveness results.

### Ethics statement

The Boston University Medical Center Institutional Review Board and the University of Zambia Biomedical Research Ethics Committee provided ethical approval of the evaluation study. This costing analysis was based on routinely collected program data, publically available information, and aggregated results from the evaluation study (e.g. proportion testing positive). All these data were obtained in anonymized form for this analysis.

## Results

### Material cost per test, material costs per dose of penicillin, training costs per test

Tables 1, 2, 3, and 4 provide basic information used to estimate the costs of syphilis testing and treatment. The cost to test one patient for syphilis is estimated at $3.10 (see Table 4), of which $1.53 is for materials, $1.03 is for labor, and $0.54 is for training (see Tables 1, 3 and 4). Training costs per health worker are estimated at $20 per month (see Table 3). With 36 new ANC patients per month on average, training costs add an additional $0.54 to syphilis diagnosis/treatment costs. Training costs would fall to about $0.23 per new ANC patient if health workers were retained longer (training needed every five years).

**Table 1:** Material costs per RST and per RPR test (ZMW and US$ 2012).

Item	Consumables	Units per test	Units per package	Cost per package (ZMW)	Cost per test (ZMW) in purchase year	Cost per test (ZMW 2012)	Cost per test (US$ 2012)
RST test-Syphilis 3.0 device	Test kit	1	30	180.00	6.00	6.00	1.15
	Gloves	2	100	11.66	0.23	0.27	0.05
	Lancet	1	200	229.21	1.15	1.32	0.25
	Alcohol	50	10.00	1	0.20	0.23	0.04
	Cotton wool	50	4.56	1	0.09	0.11	0.02
	Material costs per RST					**7.93**	**1.53**
RPR test - manual	RPR test kit	1	500	303.45	0.61	0.70	0.13
	Disposable syringe, 2 ml	1	100	5.38	0.05	0.06	0.01
	Disposable needle	1	100	5.91	0.06	0.07	0.01
	Gloves	2	100	11.66	0.23	0.27	0.05
	Alcohol	50	10.00	1	0.20	0.20	0.04
	Cotton wool	50	4.56	1	0.09	0.11	0.02
	Sterile container, 20 ml	1	25	5.45	0.22	0.25	0.05
	Materials cost per RPR					**1.66**	**0.32**

**Table 2:** Material costs per dose of penicillin (ZMW and US$ 2012).

Item	Units per package	Cost per package	Units per injection	Cost per dose (purchase year)	Cost per unit inflated to 2012 ZMK	Cost per unit (USD 2012)
Benzathine benzyl penicillin, 2.4 MU pwd for inj, one dose	10	18.02	1	1.80	2.08	0.40
Disposable syringe	100	5.38	1	0.05	0.06	0.01
Disposable needle	100	5.91	1	0.06	0.07	0.01
Gloves	100	11.66	2	0.23	0.27	0.05
Alcohol	50	10.00	1	0.20	0.20	0.04
Cotton wool	50	4.56	1	0.09	0.11	0.02
**Cost 1 Dose Penicillin**					**2.79**	**0.54**

**Table 3:** Training costs per health worker (monthly equivalent costs ZMW and US$ 2012).

	Training Cost Total (2012 ZMW)	Annual discount rate	Estimated useful life (years)	Monthly equivalent training equivalent cost (2012 ZMW)	Monthly equivalent cost (2012 USD)
Direct training costs	67,127	0.03	2	2,885	555
Salaries for trainers and participants	41,884	0.03	2	1,800	346
Total Cost				4685	901
Nurses trained				46	46
Monthly equivalent training cost per nurse (3%, 2 years)				102	20
Monthly equivalent training cost per nurse (3%, 5 years)				43	8

**Table 4:** Detailed assumptions used for base case costing and cost-effectiveness analysis (scenario ES*).

	Unit	Base case analysis	Notes
**Testing Costs**			
Materials to complete one RST	US$	1.53	See Table 1
Staff hourly salary	US$	3.55	See note**
Additional staff time per test	Hours	0.292	17.5 minutes per test from Zuznik et al.
Staff salary cost per test	US$	1.03	
Materials and staff per test	US$	2.56	
Monthly equivalent training cost per nurse	US$	19.62	See Table 3
New ANC patients per month	Patients	36.00	BUPIP records, evaluation study sites
Training cost per patient	US$	0.54	
Total cost per patient tested	US$	3.10	Materials, staff time, and training
**Treatment Costs**			
Materials for 1 dose of penicillin		0.54	See Table 2
Additional staff time per injection	US$	0.08	Authors' estimate
Additional staff salary cost per injection	Hours	0.30	
Total cost for one dose of penicillin	US$	0.83	
Proportion receiving 3 doses	US$	1.00	
**Additional Information**			
Proportion testing positive		0.028	Bonawitz et al.
Proportion patients that are true positives		0.008335	Authors' calculations***
RST sensitivity		0.98	Authors' calculations***
RST specificity		0.98	Authors' calculations***
Proportion tested		0.62	Bonawitz et al.
Proportion treated if tested positive		0.104	Bonawitz et al.
Proportion of true positives with active syphilis		0.8	Kahn et al.
Additional adverse birth outcomes due to syphilis (stillbirth/fetal loss, neonatal death)	Proportion	0.3	Gomez et al.
DALYs(3,0) per ABO attributable to syphilis		26.93	Authors calculations, 3% discount rate, no age weighting, life expectancy at birth equal to 55 years
Exchange Rate	ZMW/$	5.2	Annual average, 2012, OANDA.COM

*The base case scenario, called Scenario ES, is the full set of information that is used to estimate costs per 1,000 new ANC patients and cost effectiveness. Information in Scenario is based on prevalence and patient management as observed during the evaluation study (a 12 month period following RST training and the roll out in Kalomo District) and information on costs as presented in Tables 1–3 and additional information as needed. Sources for all information are provided in the table.

**Nurse level MS08 on government salary scale, ZMW 32,451 annual salary and all benefits, 220 working days per year, 8 hours per day.

***With 2.8% testing positive during the evaluation study, we identified the combination of true prevalence, sensitivity, and specificity that are consistent with the 2.8% testing positive.

**Table 5:** Costs per 1,000 patients presenting for antenatal care and patient management as observed during evaluation study (Scenario ES).

	Branch 1	Branch 2	Branch 3	Branch 4	Branch 5	Branch 6
	No test, Not treated	No test, treated	Tested, positive, treated	Tested, positive, not treated	Tested, negative, not treated	Tested, negative, treated
Proportion tested			0.62	0.62	0.62	0.62
Proportion not tested	0.38	0.38				
Not tested: Proportion not treated/treated	1.00	0.00				
Tested: Proportion positive			0.028	0.028		
Tested: Proportion negative					0.972	0.972
Tested: Proportion treated			0.104			0.00
Tested: Proportion not treated				0.896	1.00	
Branch probability	0.38	0.00	0.0018	0.0156	0.6026	0.00
Number of patients each branch	380.00	0.00	1.81	15.56	602.64	0.00
Diagnosis cost per branch (materials and staff)	0	0	5	40	1,535	0
Training cost per branch	207	0	1	8	328	0
Treatment cost per branch	0	0	4	0	0	0
Total cost per branch	207	0	10	48	1,871	0
Total cost per 1,000	2,136					
Total cost for testing	1,587					
Total costs of training	545					
Total costs for treatment	4.50					
**Number patients with syphilis (active or previous and cured)**						
Actual number with syphilis (active or previous and cured)	3.17	0.00	0.53	4.54	0.10	0.00
Number missed	3.17	0.00	0.00	4.54	0.10	0.00
Number correctly treated	0.00	0.00	0.53	0.00	0.00	0.00
Total number true positives (active or previous and cured)	8.33					
Total number true positives treated (active or previous and cured)	0.53					

The cost of materials per dose of penicillin is estimated at $0.54 (see Table 2). As noted in the methods section, while 3 doses are recommended, evidence suggests that one dose is adequate to reduce risks of ABOs [9]. Because the prevalence of syphilis and the material costs per dose of penicillin are both quite low, the cost of additional doses of penicillin has a very minor impact on costs (whether 1 or 3 doses are used in the costing analysis matters little). With 3 doses of penicillin and additional staff time for the injections, the cost to treat one patient is 3*$0.83 = $2.49 (Table 4).

### Costs and cost-effectiveness for each main scenario

As discussed above, we estimate costs and cost-effectiveness for three main scenarios: evaluation study conditions (ES); ES + All Positives Treated; and ES + Guidelines. We present the results for Scenario ES first (Table 5 and 6) and then discuss how these results would change with closer adherence to treatment guidelines (Table 7).

**Table 6:** Costs (USD 2012) and Cost Effectiveness (cost-per DALY avoided) per 1000 new ANC patients.

**True prevalence**	**0.008335**	
Sensitivity (SEN)	0.98	
Specificity (SPEC)	0.98	
Positive Predictive Value (PPV)	0.29	
Negative Predictive Value (NPV)	1.000	
Slide Positivity Rate RST	0.02800	
Proportion true positives (‘have syphilis’) with active syphilis	0.800	
Active syphilis cases per 1000	6.67	80% of 8.335
Cases of active syphilis per 1000 ANC patients treated	0.42	Per 1,000 patients, 0.96 patients test positive and are treated. 80% of 0.53 = 0.42 are estimated to have active syphilis.
Proportion increase in ABO (stillbirth, fetal loss, neonatal death) from active syphilis compared to no syphilis	0.30	
# ABO avoided per 1000 new ANC patients	0.13	0.42*0.303
DALYs Lost per ABO	26.93	
DALYs avoided per 1000 new ANC patients	3.40	0.13*26.93
Diagnosis and treatment costs per 1000 new ANC patients	2,136	
Cost per DALY Avoided (USD 2012)	628	2,132/3.4

**Table 7:** Summary of results per 1,000 ANC patients for main scenarios analyzed and sensitivity analyses.

Scenarios	Proportion tested with RST	Proportion treated if positive	True Prevalence*	Proportion testing positive	Cost per 1,000 new ANC patients (2012 USD)	Number of true positives identified	Number of true positives treated	DALYs avoided	Cost per DALY avoided (2012 USD)
Evaluation study (ES)	0.62	0.104	0.008335	0.028	2,136	5.06	0.53	3.40	628
ES + All Positives Treated	0.62	1.00	0.008335	0.028	2,175	5.06	5.06	32.73	66
ES + Guidelines	1.00	1.00	0.008335	0.028	3,174	8.17	8.17	52.79	60
**Sensitivity Analyses**									
2% True Prevalence									
Evaluation study (ES)	0.62	0.104	0.02	0.039	2,138	12.15	1.26	8.17	262
ES + All Positives Treated	0.62	1.00	0.02	0.039	2,192	12.15	12.15	78.54	28
ES + Guidelines	1.00	1.00	0.02	0.039	3,202	19.60	19.60	126.68	25

*True prevalence  =  proportion with active or past treated syphilis; 80% true prevalence with active syphilis.

All basic information used for Scenario ES to estimate the model (costs per 1,000 patients, cost per DALY avoided) is provided in Table 4. The main new information in Table 4, not reported in Tables 1–3 for costing or discussed previously, is the assumption that 17.5 minutes are used by a health worker to complete a test, 5 minutes are used to prepare and complete one injection, and the hourly wage for a health worker is estimated at $3.55 (details in Table 4). As reported during the evaluation study, 62% of new ANC patients were screened for syphilis using the RST. Of those screened, 2.8% tested positive, but only 10.4% were treated (received at least one dose of penicillin).

For each 1,000 patients presenting for antenatal care, the costs of syphilis diagnosis and treatment based on patient management observed during the evaluation study (Scenario ES) are estimated at $2,136 (see Table 5 and 7); $1,587 for testing; $545 for training; and only $4.50 for treatment. Table 5 also shows the basic structure of the Excel model used to estimate expected costs.

Unlike previous studies of malaria rapid diagnostic tests [12], where patients are treated if not tested and patients who test negative are nonetheless treated, deviations from guidelines during the RST evaluation study were basically the lack of testing (100% should have been tested) and the lacking of treating those testing positive (100% of those testing positive should have been treated).

In a recent general modeling exercise, it was assumed that between 90–95% of women who received a syphilis test and were found positive would actually be treated with 3 doses of penicillin [7]. Thus, the real-life situation observed during the RST roll out, where only 10.4% of positives treated, was far outside the range of situations considered in previous modeling exercises.

Of the 5.07 patients per 1,000 who are tested and test positive (active or past treated syphilis) in Scenario ES, only 1.81 are treated (Table 6). With a 29% positive predictive value and assuming that 80% of the RST positives have active syphilis, about 0.42 cases of active syphilis per 1,000 new ANC patients are treated.

With 0.42 patients with active syphilis per 1,000 new ANC patients treated, 3.40 DALYs are avoided due to the small reduction in adverse birth outcomes (see Table 6). In terms of cost effectiveness (compared to no screening or treatment), the cost-per-DALY avoided is estimated at $628.

### Impact of adherence to guidelines on costs and cost-effectiveness of RSTs

We replicated the above analysis for the two additional scenarios (ES + All positives treated; and ES + Guidelines), where all the assumptions are reported in Table 4 (and Table 7), to show how adherence to treatment guidelines affects program costs and cost effectiveness of the RST screening policy.

During the evaluation study, 62% of patients received a test, but only 10.4% were treated. Our second scenario considers the same situation if all patients testing positive were actually treated. As summarized in Table 7, program costs would remain largely the same as with scenario ES ($2,175), but DALYs avoided would increase substantially (from 6.19 to 32.73) and the cost-per-DALY avoided would fall from $628 to only $66.

If, in addition, all patients were tested and all positives treated (Scenario ES + Guidelines), program costs would rise by about 50% (from $2,136 to $3,174), but DALYs avoided would increase as well so that the cost-per-DALY avoided would fall to $60 compared to $66 for Scenario ES + All Positives Treated.

### Sensitivity analysis

Table 7 and Table 8 also report the results of sensitivity analyses for each of the three main scenarios. Cost effectiveness is fairly sensitive to the proportion of patients with active syphilis in the population. For example, with 2% true prevalence (3.9% testing positive), program costs are essentially unchanged for each scenario compared the base case, but the cost-per-DALY avoided falls by more than 50% (e.g. $628 with 0.8335% prevalence to $262 with 2% prevalence under scenario ES conditions). This number falls to just $28 if all positives and treated and $25 with adherence to guidelines (Table 7).

**Table 8:** Summary of results per 1,000 ANC patients for additional sensitivity analyses.

Sensitivity analyses	Proportion tested with RST	Proportion treated if positive	True Prevalence*	Proportion testing positive	Cost per 1,000 new ANC patients (2012 USD)	Number of true positives identified	Number of true positives treated	DALYs avoided	Cost per DALY avoided (2012 USD)
Scenario: 50% less time per test and training costs									
Evaluation study (ES)	0.62	0.104	0.008335	0.028	1,543	5.06	0.53	3.40	453
ES + All Positives Treated	0.62	1.00	0.008335	0.028	1,582	5.06	5.06	32.73	48
ES + Guidelines	1.00	1.00	0.008335	0.028	2,384	8.17	8.17	52.79	45
Scenario: 65% True Positives with Active Syphilis									
Evaluation study (ES)	0.62	0.104	0.008335	0.028	2,136	5.06	0.53	2.77	772
ES + All Positives Treated	0.62	1.00	0.008335	0.028	2,175	5.06	5.04	26.59	82
ES + Guidelines	1.00	1.00	0.008335	0.028	3,174	8.17	8.17	42.89	74
Scenario: 100% tested, 10.4% treated									
Evaluation study (ES)	1.00	0.104	0.008335	0.028	3,111	8.17	0.85	5.49	567
Scenario: 80% tested, 50%–80% treated									
Evaluation study (ES)	0.80	0.50	0.008335	0.028	2,620	6.53	3.27	21.12	124
Evaluation study (ES)	0.80	0.8	0.008335	0.028	2,637	6.53	5.23	33.79	78

*True prevalence  =  proportion with active or past treated syphilis; 80% true prevalence with active syphilis.

If staff time per test and training costs were 50% less, for example, because of better staff retention and the ability to provide other services to patients while waiting for test results, program costs would fall by about 25% for each scenario as would the cost-per-DALY avoided (Table 8). If fewer patients testing positive had active syphilis (e.g. 65%), program costs remain the same but cost-per-DALY avoided increases to $772 for Scenario ES and $82 for Scenario ES + All Positives treated (Table 8).

Table 8 also shows that achieving high levels of testing (100%) if patients found positive are not treated does little (if 10.4% of positives are treated as during the evaluation study). Costs increase by about 50%, from $2,136 for the base case (evaluation study conditions) to $3,111 and a few more DALYs are avoided (8.17 compared to 3.40), and the cost-per-DALY avoided remain high ($567). This analysis shows why it is so important, both for health outcomes and cost effectiveness, for those found positive to be treated. And last, partial but better adherence to guidelines than observed during the evaluation study (e.g. 80% testing and 50–80% of positives treated) has modest impacts on program costs as compared to Scenario ES, but cost-per-DALY avoided fall from $628 to $124–$78.

## Discussion and Conclusion

In Zambia, national guidelines recommend that all pregnant women should be tested for syphilis during their first visit for antenatal care. The primary focus of this paper was to evaluate the costs and cost-effectiveness of implementing this policy in one district of Zambia. We first estimated that the cost of syphilis screening per patient was $3.10 (see Table 1), of which $1.53 was for materials (the test kit and other minor supplies), $1.03 was for health worker time (Table 4), and $0.54 was for health worker training (Table 4). For perspective, an HIV test is also completed during the first visit for antenatal care, and the materials for an HIV test costs less than $1 [16], with a confirmatory test for those testing positive less than $2. A recent study from Southern Province of Zambia reported that the cost of antenatal and postnatal care for mother/baby pairs through six months post-partum was $31 for HIV-negative mothers and $69 for HIV-positive mothers (not including costs of antiretroviral therapy) [16]. Thus, just the materials needed for syphilis tests ($1.53) would increase antenatal/postnatal care costs by about 5% for HIV-negative mothers (the majority of women presenting for antenatal care) and about 3% for HIV-positive mothers. At the facility level, based on 36 women presenting for their visit ANC visit monthly (Table 4), syphilis testing would basically cost about $118 per month ($1416 per year if one health worker received training), of which $55 monthly is needed for test kits and minor related supplies.

We then used basic results from a related evaluation of the roll out of rapid syphilis tests in Kalomo District [5], specifically the proportion of patients receiving a test (62%), the proportion testing positive (2.8%), and the proportion treated who test positive (10.4%), as basic parameters for a cost-effectiveness analysis. For total costs (of testing and treatment), what matters most is the proportion of patients tested (because a small proportion of women will test positive and treatment is relatively inexpensive. For cost-effectiveness of syphilis screening and treatment, however, what matters most is that patients testing positive need to receive treatment. Even with very low prevalence of syphilis among new ANC patients, cost effectiveness improves dramatically if those found positive are treated because additional treatment costs little but DALYs avoided are substantial. As shown in Table 7, while the cost per DALY avoided was estimated at $628 based on actual practice during the evaluation study (62% tested but only 10.4% treated), this number would fall to just $66 if all the positives were treated with almost no impact on total costs (the budget needed to provide the service). Even with very low prevalence of active syphilis (<0.7%), with complete adherence to guidelines, where all are tested and all positives treated, the cost per DALY avoided is estimated at $60. This result is very consistent with prior modeling exercises evaluating the costs and effectiveness of syphilis testing and treatment for pregnant women, assuming reasonably good adherence to guidelines [3], [6]–[8].

In sum, while rapid syphilis tests are very good at finding the needle in a haystack, such testing only provides benefits if patients testing positive are actually treated (with at least one dose of penicillin). Otherwise, these rapid tests allow health workers to find the needle in the haystack, but the needle is then thrown back into the haystack.

Future research, which was beyond the scope of this study, needs to address the lack of adherence to guidelines and what can be done (practices, strategies, interventions) to improve adherence to syphilis testing guidelines for pregnant women. In terms of priorities, the first should be making sure that all women who are tested and test positive are actually treated, and then expanding the proportion of women who are tested. While it might be possible to address both simultaneously, the barriers for health workers to adhere to testing guidelines are likely to be different from barriers to treatment guidelines once a patient tests positive. The foundation here is commodity management. Without adequate supplies of tests, penicillin, and other related supplies, health workers cannot adhere to guidelines. When supplies exist, health workers still may not adhere which has been the case in other similar settings (e.g., rapid tests for malaria and treatment [11], [12]). A key limitation of this study is that it addresses costs and effectiveness of the introduction of rapid syphilis testing in one district (Kalomo) of one province (Southern) in Zambia during one time period. While the related evaluation study was designed to be representative to the district included in the evaluation over the evaluation period, the results cannot be used to extrapolate to other or larger geographic regions. Rather than focusing on cost-effectiveness results based on the specific assumptions in this analysis, the analytical approach (outlined in Tables 5 and 6 and provided in detail in File S2) can be used to reassess costs and cost-effectiveness in different settings with different abilities to adhere to testing and treatment guidelines.

Another limitation, as noted above in the recommendations for future research, is that this costing and cost-effectiveness study was not designed to explain why the sites did not, or could not, adhere to testing and treatment guidelines. The evaluation study addressed the first year after the roll out of the RSTs in Kalomo District as part of routine services provided during the first visit for antenatal care. An earlier evaluation during 2009–2010 of the same rapid test in two other locations in Zambia (Lusaka, a large urban area; Mongu, a rural area) found that about 96% of patients received a syphilis test during the five-month intervention period and 95% testing positive were treated [17]. At least during a few months in 2009–2010, adherence to guidelines in these locations was excellent. How to improve and sustain good adherence to testing and treatment guidelines remains an important area for future research.

## Supporting Information

File S1 **Estimating unit costs.**
(XLSX)

File S2 **The model for evaluating costs and cost effectiveness.**
(XLSX)
